# Transactions of the Forty-first Session of the American Institute of Homœopathy

**Published:** 1888-12

**Authors:** 


### Transactions of the Forty-first Session of the American Institute of Homœopathy, Niagara Falls, June, 1888. Published by Dr. Pemberton Dudley, General Secretary.

Dr. Dudley deserves great credit for publishing so promptly this neat volume of over eight hundred pages. Those who have not had any experience with such editing little appreciate the many difficulties it presents.

Of the contents of this volume, we are glad to note that they give more than usual attention to materia medica and clinical medicine. There are some papers in this volume which are really homœopathic in their tone and character. It is a welcome change. Too much attention cannot be given to study of the *Organon* and the *Materia Medica.*

